# Becoming less beige with age

**DOI:** 10.7554/eLife.89700

**Published:** 2023-07-07

**Authors:** Anying Song, Qiong A Wang

**Affiliations:** 1 Department of Molecular & Cellular Endocrinology, Arthur Riggs Diabetes and Metabolism Research Institute, City of Hope Medical Center Duarte United States; 2 Department of Molecular & Cellular Endocrinology, Arthur Riggs Diabetes and Metabolism Research Institute and the Comprehensive Cancer Center, Beckman Research Institute, City of Hope Medical Center Duarte United States

**Keywords:** adipocytes, metabolism, ageing, cold exposure, Mouse

## Abstract

The production of beige adipocytes following cold exposure is blocked as mice get older and leads to changes in the expression of metabolic genes.

**Related research article** Holman CD, Sakers AP, Calhoun RP, Cheng L, Fein EC, Jacobs C, Tsai L, Rosen ED, Seale P. 2023. Aging impairs cold-induced beige adipogenesis and adipocyte metabolic reprogramming. *eLife* **12**:RP87756. doi: 10.7554/eLife.87756.

Stored inside the fat deposits of humans and mice are thermogenic cells known as brown and beige adipocytes which burn energy and dissipate it as heat (Sakers et al., 2022). This allows the body to stay warm when temperatures drop and to maintain energy levels in response to physical activity. As individuals age, their ability to carry out this function – known as adaptive thermogenesis – declines, but it remains unclear why.

Unlike brown adipoctyes, which are located in brown adipose tissue (such as the fat deposit between shoulder blades), beige adipocytes are generated in white adipose tissue in response to certain stimuli, such as exposure to cold. The generation and activation of beige adipocytes, known as ‘beiging’, ameliorates high blood sugar (hyperglycemia) and lipid imbalances (dyslipidemia), and prevents obesity and disruption of the metabolism, making beige adipocytes a potential therapeutic target for metabolic diseases (Bartelt and Heeren, 2014; Min et al., 2016). However, beiging upon cold exposure declines with age in both rodents and humans (Berry et al., 2017; Wang et al., 2022; Benvie et al., 2023). Now, in eLife, Patrick Seale and colleagues – including Corey Holman as first author – report the results of experiments that shed light on the cellular mechanisms responsible for this age-related shift (Holman et al., 2023).

The team (who are based at the University of Pennsylvania, Beth Israel Deaconess Medical Center, Broad Institute of MIT and Harvard, and Harvard Medical School) induced beiging in the white adipose tissue in the inguinal region (also known as the groin) of mice by treating them with a drug that stimulates this process, or exposing them to cold. As expected, comparing young (9-week-old) and aged (57-week-old) mice showed that beiging was severely blunted and delayed as mice got older.

Beige adipocytes arise from adipose stem and progenitor cells via a procees known as de novo beige adipogenesis, or by reactivating dormant beige fat cells (Wang et al., 2013; Long et al., 2014; Wang et al., 2014; Vishvanath et al., 2016; Chen et al., 2019; Shao et al., 2019). Tracing the fate of adipocyte stem and progenitor cells – identified by their expression of the gene *Pdgfra* – in the white adipose tissue of reporter mice showed that aged mice produced much fewer beige adipocytes from these cells than their younger counterparts (Figure 1). This observation suggests that aging blocks de novo beige adipogenesis from adipocyte stem and progenitor cells.

**Figure 1.**
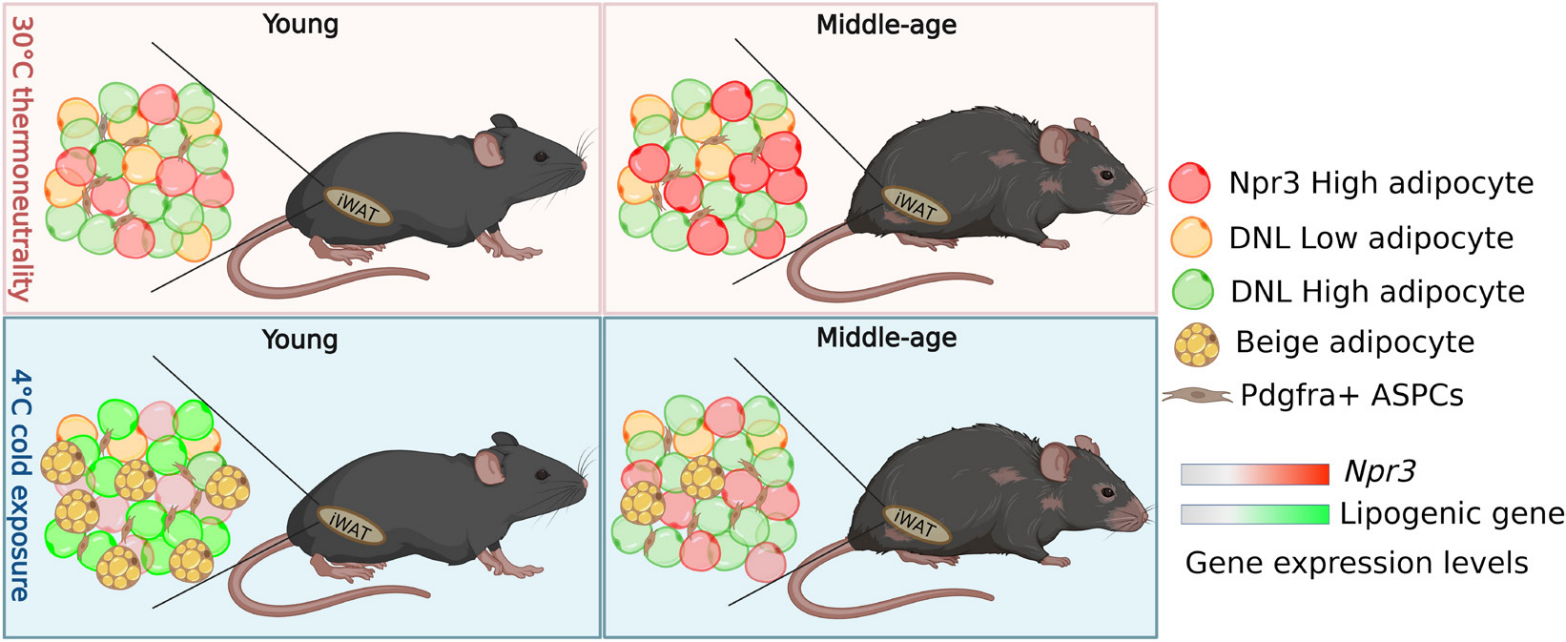
How adipocytes change in young and aged mice following cold exposure. Sitting in the white adipose tissue in the inguinal region (iWAT) of mice is a population of fat cells (represented by different coloured circles) and *Pdgfra^+^* + stem and progenitor cells (ASPCs; top left panel). When mice are exposed to cold, ASPCs mature into beige adipocytes (yellow circles with yellow spots), which burn energy and generate heat (bottom left panel). Holman et al. found that this process, known as de novo beige adipogenesis, was blocked in older mice, resulting in less beige adipocytes being generated following cold exposure (bottom right panel). Further analysis revealed three other subpopulations of adipocytes in iWAT in addition to beige adipocytes: white adipocytes expressing high levels of the thermogenic gene *Npr3* (red), and white adipocytes with low (DNL-low) or high (DNL-high) levels of de novo lipogenesis (represented as yellow and green respectively). Aging altered the proportion of DNL-low and DNL-high adipoctyes, and caused adipocytes to express higher levels of *Npr3* (top panels). Cold exposure decreased the proportion DNL-low adipoctyes in both young and older mice. It also increased the expression of lipogenic genes involved in de novo lipogenesis in young mice (bottom left panel), but not middle-aged mice (bottom right panel).

Image credit: Figure created using BioRender.

To investigate which factors contribute to this age-related decline, Holman et al. analyzed the genes expressed in individual stem and progenitor cells which had been isolated from white adipose tissue in the inguinal region. This revealed that the adipocyte stem and progenitor cells did not change their cellular identity in response to aging or cold exposure. Further analysis revealed that aging increased the expression of fibrogenic genes, such as *Cd9*, and decreased *Postn* and other extracellular matrix-related genes in adipocyte stem and progenitor cells, which may affect beige adipogenesis during aging. Moreover, Holman et al. found that adipocyte stem and progenitor cells from young and aged mice were equally competent at maturing into beige adipocytes when cultured in vitro. These results suggest that external factors surrounding these stem cells or in the bloodstream must be contributing to the age-related decline in de novo beige adipogenesis rather than cell autonomous differences.

Finally, to determine how aging and cold exposure impacts gene expression in mature adipocytes, Holman et al. performed single nuclei RNA sequencing analyses of inguinal white adipose tissue. This identified beige adipocytes and three other types of ‘white’ fat cell: adipocytes which expressed high levels of a thermogenic gene called *Npr3* that suppresses beige adipocytes from releasing energy, and adipocytes that display either high or low levels of de novo lipogenesis – the process of generating fat tissue – (named DNL-high and DNL-low, respectively; Figure 1). Aging affected the proportion of of all four adipocyte populations in white adipose tissue, and upregulated *Npr3* in the three white adipocyte populations. Interestingly, de novo lipogenesis induced by cold exposure was severely impaired in the beige and DNL-high adipocytes of aged mice. These results suggest that the dysregulation of signaling pathways, such as *Npr3* signaling and lipogenesis, during aging may contribute to declining beige adipogenesis.

Senescence of adipocyte progenitor cells (Berry et al., 2017; Benvie et al., 2023) and inactivation of thermogenic genes in mature fat cells (Wang et al., 2022) have already been associated with the age-related impairment of beiging. Here, Holman et al. provide direct in vivo evidence that de novo beige adipogenesis is also blocked during aging in mice, revealing another possible mechanism to explain age-related reductions in beige adipogenesis. This work also offers a unique resource for researchers who are trying to identify the signaling pathways related to reactivating dormant beige adipocytes.
